# High prevalence of *Helicobacter pylori* mixed infections identified by multilocus sequence typing in Ningbo, China

**DOI:** 10.3389/fmicb.2023.1207878

**Published:** 2023-08-08

**Authors:** Yongxiong Zhang, Haiying Gu, Zhouhong Shi, Weiqin Chen, Airu Li, Weiwei Ye, Cheng Zhang, Huikun Yuan, Mingming Zhao

**Affiliations:** ^1^Health Science Center, Ningbo University, Ningbo, Zhejiang, China; ^2^Laboratory of Gastroenterology, The Affiliated Hospital of Medical School, Ningbo University, Ningbo, Zhejiang, China; ^3^Department of Gastrology, Ninghai First Hospital, Ningbo, Zhejiang, China

**Keywords:** *Helicobacter pylori*, mixed infections, unrelated strains, evolutionary relationships, homologous, multilocus sequence typing, phylogeographic groups

## Abstract

This study used multilocus sequence typing (MLST) to investigate the prevalence of *Helicobacter pylori* (*H. pylori*) mixed infections and *H. pylori* mixed infections involving unrelated strains; and determined the phylogeographic groups of *H. pylori* recovered from patients in Ningbo, China. A total of 156 *H. pylori* isolates were obtained from a convenience sample of 33 patients with culture-positive *H. pylori* infection. MLST was used to classify 150 *H. pylori* clinical isolates and 12 methodological control strains (6 clinical isolates and 6 strains of American Type Culture Collection *H. pylori*) into 43 and 12 sequence types (STs), respectively. In this study, 246 new alleles and 53 new STs were identified by MLST. The prevalence of mixed infections was 41% (11/27). The prevalence of *H. pylori* mixed infections involving unrelated strains was 46% (5/11) and the prevalence of *H. pylori* mixed infections involving completely unrelated strains (strains with all 7 housekeeping genes different) was 36% (4/11). A phylogenetic tree was created to determine the evolutionary relationships between different strains. The STs in this study were clustered within the hspEAsia subgroup (98%) and hpEurope group (2%). *H. pylori* mixed infections were common in Ningbo, China. The *H. pylori* isolates belonging to the hpEurope group were recovered from three different biopsy samples in a native Chinese patient. Most of *H. pylori* strains colonizing the antrum, corpus, and duodenum bulb were homologous.

## Introduction

*Helicobacter pylori* (*H. pylori*) is a human pathogen that colonizes the gastrointestinal mucosa and causes chronic active gastritis, peptic ulcers, lymphoid tissue lymphoma, and gastric adenocarcinoma (Blaser and Atherton, 2004; Burkitt et al., 2017; Camilo et al., 2017). It is a gram-negative microaerophilic bacterium and has a high prevalence worldwide.

The gastrointestinal mucosa can be colonized by single or multiple *H. pylori* strains (Blaser and Berg, 2001; Kusters et al., 2006; Talebi Bezmin Abadi and Perez-Perez, 2016). *H. pylori* mixed infections and heteroresistance may lead to the failure of eradication treatment, based on different resistance of isolates from the antrum and corpus to antimicrobial agents (Farzi et al., 2019). *H. pylori* mixed infections are a cause for concern. The prevalence of *H. pylori* mixed infections varies from 0 to 100% in different geographic regions (Ben Mansour et al., 2016). The evolutionary relationships between different *H. pylori* isolates recovered from individual hosts are unclear. Previous studies have described four patterns of mixed infection: (i) most *H. pylori* isolates are related and exhibit slightly different patterns, and only few isolates are unrelated (Kim et al., 2003; Carroll et al., 2004; Kao et al., 2014; Farzi et al., 2015; Mendoza-Elizalde et al., 2019); (ii) all isolates are related (Patra et al., 2012; Ren et al., 2012; Kibria et al., 2015); (iii) isolates are predominantly unrelated (70%), with a few (30%) related isolates exhibiting slightly different patterns (Seo et al., 2019); (iv) all isolates are unrelated or show two independent populations of *H. pylori* (Carroll et al., 2004; Matteo et al., 2007; Salama et al., 2007). The prevalence of mixed infections varies according to the geographic region and the participant selection criteria (Kersulyte et al., 2000; Wong et al., 2001; Ben Mansour et al., 2016). *H. pylori* isolates with slightly different DNA fingerprinting patterns from a single host have revealed the mechanisms of microevolution (Marshall et al., 1998; Kim et al., 2003; Carroll et al., 2004; Mendoza-Elizalde et al., 2019). Due to its limitations, the DNA fingerprinting method cannot determine the evolutionary relationships of isolates from a single host (Carroll et al., 2004; Matteo et al., 2007).

Multilocus sequence typing (MLST) is based on comparison of the sequences of seven housekeeping genes (*atpA*, *efp*, *mutY*, *ppa*, *trpC*, *ureI*, and *yphC*) (Osaki et al., 2015; Raaf et al., 2017; Mendoza-Elizalde et al., 2019) and has the advantages of high repeatability, high resolution, and normalization. Moreover, this method can be used to determine the evolutionary relationships of isolates recovered from a single patient with *H. pylori* mixed infections (Mendoza-Elizalde et al., 2019). The prevalence of *H. pylori* mixed infections involving strains that are unrelated according to MLST is unclear. Importantly, the geographical location of the human host can be inferred from the MLST results (Mégraud et al., 2016; Gutiérrez-Escobar et al., 2017; Vazirzadeh et al., 2022). However, there is a lack of data on the geographic grouping of *H. pylori* isolates from patients in southern urban China.

Therefore, the purpose of this study was to characterize *H. pylori* mixed infections by MLST. More specifically, we investigated the prevalence of *H. pylori* mixed infections involving unrelated strains and phylogeographic groups of *H. pylori* isolates recovered from single patients in Ningbo, China.

## Materials and methods

### Ethics statement

Ethical approval for this study was obtained from the Medical Research Committee of the Ningbo University School of Medicine (approval number: NBU-2021-129).

### Patients and specimens

A convenience sample of 33 patients from the Ninghai First Hospital and the Affiliated Hospital of Ningbo University School of Medicine who were culture positive for *H. pylori* participated in the study. Patients were excluded if they had taken antibiotics, proton pump inhibitors, H2-receptor antagonists, non-steroidal anti-inflammatory drugs, or bismuth-containing compounds within the 15 days prior to endoscopy. All patients provided written informed consent to participate in the study. Six patients were assigned to the control group, in which the prevalence of *H. pylori* mixed infections was not compared. Two or three mucosal biopsy specimens were collected from the antrum, corpus, and/or duodenal bulb of each patient for bacterial culture. The specimens were immediately placed in a transport medium (Gu’s Kit for Preservation of *Helicobacter pylori*, TianKuo, Ningbo Xunjian Biotechnology Co., Ltd., Ningbo, China) and transported to the laboratory at 2–8°C. Two other biopsy specimens were collected from each patient for histological examination.

### Isolation and identification of *H. pylori*

The biopsy specimens were homogenized and inoculated on Gu’s plates (Gu’s Medium for Rapid Isolation of *Helicobacter pylori*, TianKuo, Ningbo Xunjian Biotechnology Co., Ltd., Ningbo, China; PCT WO2022178982A1). The plates were incubated at 37°C for 2–5 days in a microaerophilic environment (3–5% O_2_, 5–10% CO_2_, 5–10% H_2_, 75–87% N_2_) with 100% humidity (Anoxomat; MART Microbiology BV, Drachten, Netherlands). A pool of colonies was selected from the primary culture plates for subculturing. Single colonies were obtained from biopsy specimens of different sites of each patient using the colony suspension dilution method, and 1–10 single colonies were selected and passaged separately to obtain single colony isolates.

One of the three isolation schemes a, b, and c was selected to analyze *H. pylori* mixed infections in each patient, as follows: (a) One *H. pylori* colony was isolated from each of multiple biopsy specimens of a single patient; (b) Multiple (3–11) single colonies were obtained from one biopsy specimen of a single patient; (c) Multiple (2–10) single colonies were isolated from each of several biopsy specimens of a single patient.

*Helicobacter pylori* was identified based on colony morphology and Gram staining as a gullwing-shaped bacterium; positive reactions for catalase, oxidase, and urease (Gu’s Kit for Rapid Identification of *Helicobacter pylori*, TianKuo, Ningbo Xunjian Biotechnology Co., Ltd., Ningbo, China); and *H. pylori* antigen testing (*H. pylori* Antigen Rapid Test, Abon Biopharm, Hangzhou, China). Six American Type Culture Collection (ATCC) *H. pylori* reference strains (ATCC 43629, ATCC 700392, ATCC 51932, ATCC 700824, ATCC 43579, and ATCC 49503) were used as controls.

### DNA extraction

Genomic DNA was extracted using a HiPure Bacterial DNA Kit (Guangzhou Magen Biotechnology Co., Ltd., Guangzhou, China) according to the manufacturer’s instructions. The extracted genomic DNA was stored at −20°C until being amplified using polymerase chain reaction (PCR).

### Multilocus sequence typing

The extracted DNA was used as a template for PCR amplification of seven housekeeping genes (*atpA*, *efp*, *mutY*, *ppa*, *trpC*, *ureI*, and *yphC*). The primers used for the MLST housekeeping genes are shown in Table 1. The PCR amplification conditions used were as described by Achtman et al. (1999). The PCR products were detected, purified, and sequenced using Sanger sequencing (QingKe Biotechnology Co., Ltd., Hangzhou, China). The sequence peaks were interpreted using Chromas software to remove miscellaneous peaks, and the resulting sequences were imported into DNA Star software and assembled using the SeqMan function. The trimmed sequences of the seven housekeeping genes were imported into the *H. pylori* PubMLST database^1^ to identify allelic matches. The STs were identified based on the combination of MLST alleles. Nucleotide sequences that did not match existing PubMLST sequences were submitted to the database for new number assignment and sequence typing.

**TABLE 1:** Primers for the multilocus sequence typing (MLST) housekeeping gene.

Gene	Primer sequence (5′→3′)	Product size (bp)	Annealing temperature
*atpA*	*atpA*-F: GGACTAGCGTTAAACGCACG *atpA*-R: CTTGAAACCGACAAGCCCAC	627	55°C
*efp*	*efp*-F: GGCAATTTGGATGAGCGAGCTC *efp*-R: CTTCACCTTTTCAAGATACTC	410	55°C
*mutY*	*mutY*-F: GTGGTTGTAGYTGGAAACTTTACAC *mutY*-R: CTTAAGCGTGTGTYTTTCTAGG	420	55°C
*ppa*	*ppa*-F: GGAGATTGCAATGAATTTAGA *ppa*-R: GTGGGGTTAARATCGTTAAATTG	398	55°C
*trpC*	*trpC*-F: TAGAATGCAAAAAAGCATCGCCCTC *trpC*-R: TAAGCCCGCACACTTTATTTTCGCC	456	55°C
*ureI*	*ureI*-F: AGGTTATTCGTAAGGTGCG *ureI*-R: GTTTAAATCCCTTAGATTGCC	585	55°C
*yphC*	*yphC*-F: CACGCCTATTTTTTTGACTAAAAAC *yphC*-R: CATTYACCCTCCCAATGATGC	510	55°C

*atpA*, encodes an ATP synthase alpha chain; *efp*, encodes an elongation factor P; *mutY*, encodes a DNA glycosylase; *ppa*, encodes an inorganic pyrophosphatase; *trpC*, encodes an anthranilate isomerase; *ureI*, encodes a urease subunit I; *yphC*: encodes a GTPase.

### Phylogeny, genealogic analysis, and genetic diversity analysis

The genealogic relationships among *H. pylori* isolates from the patients with mixed infections were investigated using PHYLOViZ (Mendoza-Elizalde et al., 2019). The goeBURST algorithm was used to demonstrate the relationship between clonal complexes per patient at the triple locus variants (TLV) level. The neighbor-joining algorithm with the Hamming distance was utilized to calculate the phylogenetic distance among strains using the Saitou and Nei (1987) criterion.

A phylogenetic tree was constructed using the MEGA 11 software for evolutionary analysis (Tamura et al., 2021). The concatenated nucleotide sequences of seven housekeeping genes in the studied *H. pylori* clinical isolates and six reference strains (ATCC 43629, ATCC 700392, ATCC 51932, ATCC 700824, ATCC 43579, and ATCC 49503) were aligned in ClustalW. Phylogenetic distances were computed using the neighbor-joining method (Saitou and Nei, 1987) with the Kimura 2-parameter model of nucleotide substitution (Kimura, 1980). The reliability of clustering was assessed using a bootstrap test (1,000 bootstrap replications). Polymorphisms in the housekeeping genes of *H. pylori* isolates obtained from patients with mixed infections were analyzed using DnaSP v6, which included polymorphic sites (S), nucleotide diversity (Pi), number of haplotypes (h), and haplotype diversity (Hd).

In addition, we downloaded 284 concatenated nucleotide sequences of the seven housekeeping genes of *H. pylori* strains from the PubMLST database^2^ as representatives of different geographical groups to determine the geographical type of *H. pylori* strains obtained in this study. After aligning the concatenated nucleotide sequences of the seven housekeeping genes in the studied strains (55 STs) and reference sequences (284 STs) by ClustalW, a phylogenetic tree was constructed in MEGA 11. The reference sequences for the geographical groups were as follows: hpEurope, 75 sequences; hpAsia2, 18 sequences; hspMaori, 35 sequences; hspEAsia, 50 sequences; hspAmerind, 9 sequences; hpAfrica2, 3 sequences; hspSAfrica, 25 sequences; hspWAfrica, 29 sequences; hpNEAfrica, 20 sequences; and hpSahul, 20 sequences.

## Results

### *H. pylori* isolation

A total of 156 clinical isolates of *H. pylori* were obtained from 33 patients with dyspepsia (18 males and 15 females) at two hospitals: Ninghai First Hospital and the Affiliated Hospital of Ningbo University School of Medicine. Clinical data showed that among the 33 patients with gastric diseases investigated by gastric endoscopy, 6 (18%) had gastric ulcer, 4 (12%) had duodenal ulcer, and 2 (6%) had compound ulcer, whereas the remaining 13 (39%), 7 (21%), and 1 (3%) were diagnosed with chronic active gastritis, chronic superficial gastritis, and chronic atrophic gastritis by pathology, respectively. The clinical data of the 33 patients and information on the sampling of *H. pylori* isolates and the number of isolates are summarized in Supplementary Table 1.

### Multilocus sequence typing results

Multilocus sequence typing (MLST) was used to classify 162 *H. pylori* strains into 55 sequence types (STs). The STs of the six *H. pylori* isolates in the clinical control group were ST3689, ST3744, ST4096, ST4097, ST3715, and ST3696. The STs of the six ATCC reference strains were ATCC 43629 for ST4089, ATCC 700392 for ST181, ATCC 51932 for ST4091, ATCC 700824 for ST3496, ATCC 43579 for ST4092, and ATCC 49503 for ST4095. MLST separated the six clinical control isolates and the six ATCC reference strains into twelve STs. This indicated that isolates from different patients possessed different STs and that MLST genotyping in this study was accurate. The STs of 156 *H. pylori* isolates from the 33 patients as well as 6 ATCC strains are shown in Table 2. The analysis of seven housekeeping genes in these strains revealed 246 new alleles (*atpA*, 39; *efp*, 37; *mutY*, 37; *ppa*, 23; *trpC*, 38; *ureI*, 37; *yphC*, 35) and 53 new STs.

**TABLE 2:** Dataset of alleles and STs of *Helicobacter pylori* in this study.

	Isolation source	ST	Allele						
			***atpA***	***efp***	***mutY***	***ppa***	***trpC***	***ureI***	***yphC***
ATCC 43629		4,089	198	92	234	222	226	306	317
ATCC 700392		181	181	181	181	181	181	181	181
ATCC 51932		4,09	1,749	200	1,803	201	1,828	3,322	2,059
ATCC 700824		3,496	2,762	199	2,806	199	199	199	199
ATCC 43579		4,092	3,189	2,992	3,280	3,040	3,318	3,323	3,397
ATCC 49503		4,095	3,190	2,993	3,284	3,041	3,319	3,324	3,398
Patient 1	DB	3,689	2,886	2,697	2,945	2,774	2,996	2,992	3,063
Patient 2	DB	3,744	2,915	2,726	2,972	253	3,025	3,019	3,088
Patient 3	A	4,096	3,192	2,995	3,282	3,043	3,321	3,327	3,400
Patient 4	C	4,097	3,193	2,996	3,283	3,043	3,322	3,326	3,401
Patient 5	A	3,715	2,906	2,723	2,966	2,781	3,020	3,015	3,083
Patient 6	DB	3,696	2,895	2,697	2,945	2,774	2,996	2,992	3,063
Patient 7	A	3,710	2,903	2,699	2,947	2,768	2,998	2,994	3,065
	C	3,692	2,887	1,760	2,947	2,768	3,003	2,994	3,065
	DB	4,090	2,887	2,699	2,947	2,768	2,998	2,994	3,065
Patient 8	A	3,735	2,889	2,727	2,976	2,783	3,029	3,023	3,093
	C	3,735	2,889	2,727	2,976	2,783	3,029	3,023	3,093
	DB	3,735	2,889	2,727	2,976	2,783	3,029	3,023	3,093
Patient 9	C	3,731	2,888	2,700	2,960	2,770	2,999	2,995	3,066
	DB	3,731	2,888	2,700	2,960	2,770	2,999	2,995	3,066
Patient 10	A	3,734	2,917	1,892	2,973	448	3,026	3,020	3,090
	C	3,733	2,903	1,892	2,973	448	3,026	3,020	3,090
	DB	3,734	2,917	1,892	2,973	448	3,026	3,020	3,090
Patient 11	A	4,093	2,890	2,701	2,948	2,769	3,000	2,996	3,398
	C	4,094	3,191	2,994	3,281	3,042	3,320	3,325	3,071
Patient 12	C	3,693	2,891	2,702	2,949	2,391	3,001	1,069	3,063
	DB	3,693	2,891	2,702	2,949	2,391	3,001	1,069	3,063
Patient 13	A	3,699	1,987	1,870	2,961	2,415	3,008	3,002	3,071
	C	3,699	1,987	1,870	2,961	2,415	3,008	3,002	3,071
	DB	3,699	1,987	1,870	2,961	2,415	3,008	3,002	3,071
Patient 14	C	3,694	2,892	2,703	2,950	2,770	3,002	2,997	3,067
Patient 15	DB	3,736	2,919	2,728	2,977	2,784	3,030	3,024	3,094
Patient 16	A	3,695	2,894	2,705	2,951	6	3,004	2,998	3,068
	C	3,695	2,894	2,705	2,951	6	3,004	2,998	3,068
	DB	3,695	2,894	2,705	2,951	6	3,004	2,998	3,068
Patient 17	A	3,732	2,916	2,698	2,946	2,767	2,997	2,993	3,064
	C	3,709	2,908	2,698	2,946	2,767	2,997	2,993	3,064
Patient 18	A	3,697	2,903	2,704	2,952	2,771	3,006	3,000	3,069
	C	3,697	2,903	2,704	2,952	2,771	3,006	3,000	3,069
	DB	3,697	2,903	2,704	2,952	2,771	3,006	3,000	3,069
Patient 19	A	3,711	2,896	2,706	2,956	2,772	3,007	3,001	3,075
	C	3,698	2,896	2,706	2,956	2,772	3,007	3,001	3,070
	DB	3,698	2,896	2,706	2,956	2,772	3,007	3,001	3,070
Patient 20	A	3,706	2,902	2,712	2,010	2,776	3,013	1,364	3,079
	C	3,706	2,902	2,712	2,010	2,776	3,013	1,364	3,079
	DB	3,747	2,902	2,712	2,978	2,776	3,013	1,364	3,079
Patient 21	A	3,700	2,897	2,707	2,953	448	3,009	3,003	3,072
		3,701	2,903	2,707	2,953	448	3,009	3,003	3,072
	DB	3,700	2,897	2,707	2,953	448	3,009	3,003	3,072
		3,701	2,903	2,707	2,953	448	3,009	3,003	3,072
		3,702	2,898	2,707	2,953	448	3,009	3,003	3,072
Patient 22	A	3,704	2,899	2,710	2,954	1,279	3,010	3,004	3,073
		3,746	2,903	2,710	2,954	1,279	3,010	3,004	3,073
	C	3,723	2,913	2,714	2,955	2,773	3,011	3,005	3,074
Patient 23	A	3,737	2,920	2,708	2,957	2,775	3,013	3,007	3,076
	C	3,737	2,920	2,708	2,957	2,775	3,013	3,007	3,076
	DB	3,737	2,920	2,708	2,957	2,775	3,013	3,007	3,076
Patient 24	A	3,705	2,901	2,711	2,958	932	3,014	3,008	3,077
	C	3,705	2,901	2,711	2,958	932	3,014	3,008	3,077
	DB	3,705	2,901	2,711	2,958	932	3,014	3,008	3,077
Patient 25	A	3,738	2,900	2,716	2,963	2,778	3,017	3,018	3,095
	C	3,738	2,900	2,716	2,963	2,778	3,017	3,018	3,095
	DB	3,738	2,900	2,716	2,963	2,778	3,017	3,018	3,095
Patient 26	A	3,739	2,904	2,721	2,964	2,779	3,018	3,017	3,081
	C	3,739	2,904	2,721	2,964	2,779	3,018	3,017	3,081
	DB	3,739	2,904	2,721	2,964	2,779	3,018	3,017	3,081
Patient 27	A	3,730	2,905	2,722	2,965	2,780	3,019	3,016	3,082
	C	3,730	2,905	2,722	2,965	2,780	3,019	3,016	3,082
Patient 28	C	3,740	2,893	2,709	2,962	502	3,005	3,010	3,080
	DB	3,740	2,893	2,709	2,962	502	3,005	3,010	3,080
Patient 29	A	3,708	2,903	2,713	2,959	2,777	3,015	3,009	3,078
	C	3,708	2,903	2,713	2,959	2,777	3,015	3,009	3,078
Patient 30	A	3,704	2,899	2,710	2,954	1,279	3,010	3,004	3,073
	C	3,723	2,913	2,714	2,955	2,773	3,011	3,005	3,074
Patient 31	A	3,716	2,907	2,715	2,967	s499	3,012	3,014	3,084
	C	3,716	2,907	2,715	2,967	499	3,012	3,014	3,084
		3,741	2,910	2,725	2,968	2,785	3,021	3,013	3,085
		3,718	2,911	2,724	2,969	2,782	3,022	3,012	3,086
Patient 32	A	3,748	2,909	2,717	2,982	56	3,023	3,011	3,087
Patient 33	A	3,720	963	2,718	2,970	7	3,016	2,999	3,089
		3,724	963	2,718	2,970	7	3,016	3,006	3,089
		3,743	2,912	2,719	2,970	1,466	3,024	3,006	3,089
		3,749	2,922	2,718	2,970	7	3,016	2,999	3,089
	C	3,720	963	2,718	2,970	7	3,016	2,999	3,089
		3,742	963	2,720	2,970	7	3,016	2,999	3,089

The newly emerged alleles and STs of the isolates from this study are indicated in red. A, antrum; C, corpus; DB, duodenal bulb.

### Evolutionary relationships between isolates in patients with mixed infections

**FIGURE 1:**
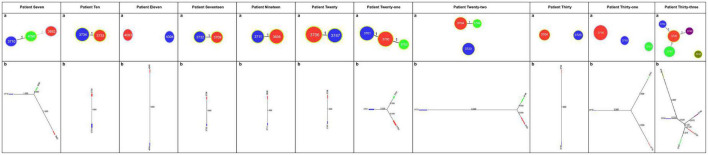
Evolutionary relationships among STs of *H. pylori* in patients with mixed infections. **(A)** Image showing the clonal relationships between the STs of *H. pylori*. Each circle represents an ST, and the size of the circle indicates the number of clinical isolates in the ST. The number on each line indicates the number of alleles with mutations. The unlinked STs imply that there are more than three different housekeeping genes between them. PHYLOViZ (goeBURST algorithm) was used to define the clonal relationships. **(B)** Image showing the evolutionary distance between the STs of *H. pylori*. Each square represents an ST; the larger the square, the more clinical isolates are included. Moreover, the longer the line and the larger the value on the line, the greater the genetic distance. PHYLOViZ (neighbor-joining algorithm) was used to define the evolutionary distance.

The different STs identified in each patient with mixed infections were used as genotyping data for the PHYLOViZ platform (Mendoza-Elizalde et al., 2019). The evolutionary relationships between isolates in the 11 patients with mixed infections are shown in Figure 1. The goeBURST algorithm was used at the TLV level to define clonal relationships. In the case of Patient 7, ST4090 was the major clonal complex with two linked STs (ST3710 and ST3692). The neighbor-joining algorithm was used to define the evolutionary distance; the longer the line, the greater the genetic distance. The number on the line represents how many housekeeping genes differ between these STs. For example, Patient 7 had one different housekeeping gene between ST3710 and ST4090, two differences between ST4090 and ST3692, and three differences between ST3710 and ST3692.

According to the evolutionary relationships and the polymorphisms of housekeeping genes of these isolates (Table 3), four of the eleven patients with mixed infections had isolates with seven different housekeeping genes, among which three patients (Patients 11, 22, and 30) showed different STs colonized at different sites (antrum, corpus), and one patient (Patient 31) exhibited three different STs at the same site (corpus). Moreover, two patients (Patients 7 and 33) had isolates with up to three and five different housekeeping genes, respectively. Eight of the eleven patients had isolates with only one housekeeping gene change, with *atpA* accounting for 75% (6/8), *mutY* for 12.5% (1/8) and *yphC* for 12.5% (1/8). Based on clonal relationships at the TLV level, related strains had the same STs or STs that differed by only very few alleles, whereas unrelated strains had STs that differed by more than three alleles. Therefore, the isolates from five patients (Patients 11, 22, 30, 31 and 33) were considered unrelated strains, and even isolates from four of these patients were considered completely unrelated strains (strains with all seven housekeeping genes different).

**TABLE 3:** Polymorphisms of housekeeping genes of *Helicobacter pylori* isolates obtained from 11 patients with mixed infections.

	Gene	Polymorphic sites (S)	Number of haplotypes (h)	Haplotype diversity (Hd)	Nucleotide diversity (Pi)
Patient 7	*atpA*	6	2	0.667	0.00638
	*efp*	4	2	0.667	0.00650
	*mutY*	0	1	0.000	0.00000
	*ppa*	0	1	0.000	0.00000
	*trpC*	1	2	0.667	0.00146
	*ureI*	0	1	0.000	0.00000
	*yphC*	0	1	0.000	0.00000
	Concatenated	11	3	1.000	0.00215
Patient 10	*atpA*	9	2	0.667	0.00957
	*efp*	0	1	0.000	0.00000
	*mutY*	0	1	0.000	0.00000
	*ppa*	0	1	0.000	0.00000
	*trpC*	0	1	0.000	0.00000
	*ureI*	0	1	0.000	0.00000
	*yphC*	0	1	0.000	0.00000
	Concatenated	9	2	0.667	0.00176
Patient 11	*atpA*	16	2	1.000	0.02552
	*efp*	11	2	1.000	0.02683
	*mutY*	17	2	1.000	0.04048
	*ppa*	6	2	1.000	0.01508
	*trpC*	116	2	1.000	0.25439
	*ureI*	6	2	1.000	0.01026
	*yphC*	19	2	1.000	0.03725
	Concatenated	191	2	1.000	0.05608
Patient 17	*atpA*	3	2	1.000	0.00478
	*efp*	0	1	0.000	0.00000
	*mutY*	0	1	0.000	0.00000
	*ppa*	0	1	0.000	0.00000
	*trpC*	0	1	0.000	0.00000
	*ureI*	0	1	0.000	0.00000
	*yphC*	0	1	0.000	0.00000
	Concatenated	3	2	1.000	0.00088
Patient 19	*atpA*	0	1	0.000	0.00000
	*efp*	0	1	0.000	0.00000
	*mutY*	0	1	0.000	0.00000
	*ppa*	0	1	0.000	0.00000
	*trpC*	0	1	0.000	0.00000
	*ureI*	0	1	0.000	0.00000
	*yphC*	15	2	0.667	0.01961
	Concatenated	15	2	0.667	0.00294
Patient 20	*atpA*	0	1	0.000	0.00000
	*efp*	0	1	0.000	0.00000
	*mutY*	1	2	0.667	0.00159
	*ppa*	0	1	0.000	0.00000
	*trpC*	0	1	0.000	0.00000
	*ureI*	0	1	0.000	0.00000
	*yphC*	0	1	0.000	0.00000
	Concatenated	1	2	0.667	0.00020
Patient 21	*atpA*	8	3	0.581	0.00673
	*efp*	0	1	0.000	0.00000
	*mutY*	0	1	0.000	0.00000
	*ppa*	0	1	0.000	0.00000
	*trpC*	0	1	0.000	0.00000
	*ureI*	0	1	0.000	0.00000
	*yphC*	0	1	0.000	0.00000
	Concatenated	8	3	0.581	0.00124
Patient 22	*atpA*	13	3	0.600	0.00787
	*efp*	14	2	0.533	0.01821
	*mutY*	12	2	0.533	0.01524
	*ppa*	7	2	0.533	0.00938
	*trpC*	14	2	0.533	0.01637
	*ureI*	15	2	0.533	0.01368
	*yphC*	13	2	0.533	0.01359
	Concatenated	88	3	0.600	0.01319
Patient 30	*atpA*	8	2	0.485	0.00619
	*efp*	14	2	0.485	0.01656
	*mutY*	12	2	0.485	0.01385
	*ppa*	7	2	0.485	0.00853
	*trpC*	14	2	0.485	0.01489
	*ureI*	15	2	0.485	0.01243
	*yphC*	13	2	0.485	0.01236
	Concatenated	83	2	0.485	0.01182
Patient 31	*atpA*	15	3	0.242	0.00368
	*efp*	11	3	0.242	0.00467
	*mutY*	16	3	0.242	0.00760
	*ppa*	7	3	0.242	0.00247
	*trpC*	19	3	0.242	0.00592
	*ureI*	8	3	0.242	0.00208
	*yphC*	16	3	0.242	0.00606
	Concatenated	92	3	0.242	0.00452
Patient 33	*atpA*	4	3	0.205	0.00082
	*efp*	3	3	0.526	0.00163
	*mutY*	0	1	0.000	0.00000
	*ppa*	1	2	0.105	0.00026
	*trpC*	5	2	0.105	0.00115
	*ureI*	1	2	0.199	0.00034
	*yphC*	0	1	0.000	0.00000
	Concatenated	14	5	0.649	0.00059

**FIGURE 2:**
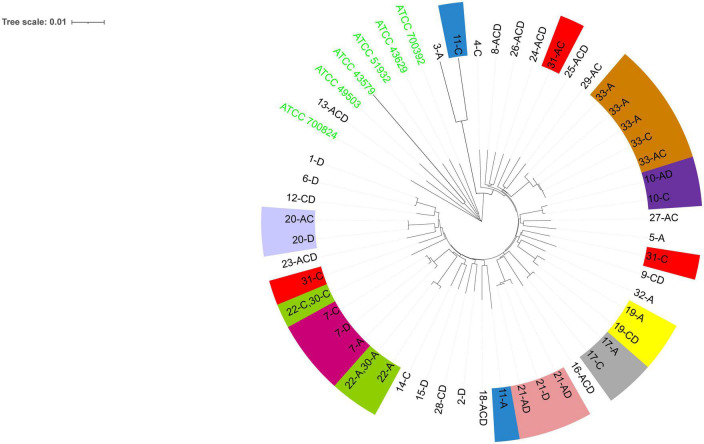
The evolutionary history was inferred using the neighbor-joining method (Saitou and Nei, 1987). The optimal tree is shown. The evolutionary distances were computed using the Kimura (1980) 2-parameter method and were in the units of the number of base substitutions per site. This analysis involved 55 nucleotide sequences. All positions containing gaps and missing data were eliminated (complete deletion option). A total of 3,406 positions were included in the final dataset. Evolutionary analyses were conducted using MEGA11 (Tamura et al., 2021). The six ATCC strains shown are the reference strains. Numbers 1–33 are patient numbers. Strains from the same patient are indicated by the same color. A, antrum; C, corpus; D, duodenum bulb.

Phylogenetic analysis (Figure 2) showed that among the patients with mixed infections, the strains from four patients (Patients 11, 22, 30, and 31) were genetically distant and did not cluster together, indicating no evolutionary relatedness. The seven housekeeping genes of the isolates from these four patients were all different.

### Phylogeography

**FIGURE 3:**
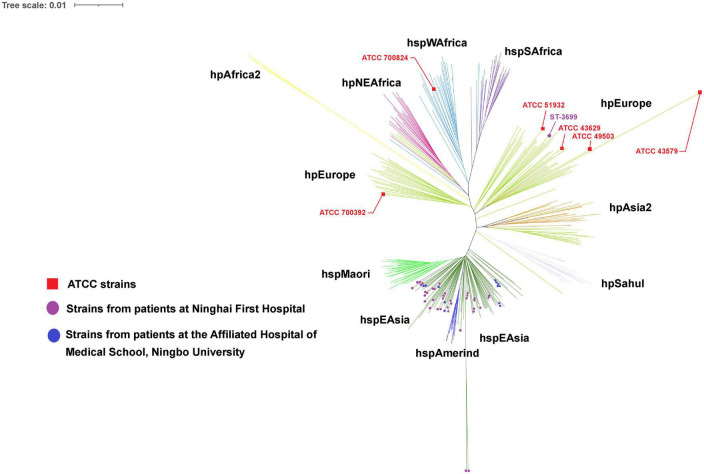
Phylogeography of the analyzed strains. The phylogeography was inferred using the neighbor-joining method (Saitou and Nei, 1987). The optimal tree is shown. The evolutionary distances were computed using the Kimura (1980) 2-parameter method and were in the units of the number of base substitutions per site. This analysis involved 55 (in this study) + 284 [from the *Helicobacter pylori* PubMLST database (http://pubmlst.org/organisms/helicobacter-pylori)] nucleotide sequences. All positions containing gaps and missing data were eliminated (complete deletion option). A total of 3,406 positions were included in the final dataset. Phylogeographical analyses were conducted using MEGA11 (Tamura et al., 2021).

The phylogenetic tree revealed that the majority (153) of the 156 *H. pylori* clinical isolates belonged to the hspEAsia subgroup, whereas the three isolates (ST3699) from Patient 13 belonged to the hpEurope group (Figure 3). Among the reference strains, ATCC 700824 belonged to hspWAfrica, and the rest belonged to hpEurope.

## Discussion

*Helicobacter pylori* is the most common infectious pathogen causing gastrointestinal diseases in humans, and can cause chronic gastritis, peptic ulcer, and gastric cancer (Blaser and Atherton, 2004; Burkitt et al., 2017; Camilo et al., 2017). Individuals can be infected by one or more types of *H. pylori*, which is known as multiple infections (Didelot et al., 2013; Palau et al., 2020), co-infection (Didelot et al., 2013; Seo et al., 2019; Mi et al., 2021), or mixed infections (Kao et al., 2014; Lai et al., 2016). Ben Mansour et al. (2016) distinguished the concepts of multiple infections and mixed infections, and defined multiple infections as an individual with two or more genetically different *H. pylori* isolates, whereas they defined mixed infections as an individual with two or more *H. pylori* isolates with different antibiotic sensitivity characteristics. It would contradict much of the literature to define mixed infections as simply different strains involving differences in antibiotic susceptibility characteristics. In essence, mixed infections are the colonization of the upper gastrointestinal mucosa of an individual by strains with heterogeneous biological characteristics, which can be reflected in the phenotypic characteristics of the strains, such as virulence factors, heteroresistance (Lai et al., 2016; Mi et al., 2021), and as well as strains with heterogeneous DNA.

The diagnosis of *H. pylori* mixed infection is mainly through genotyping techniques, including detection of virulence genes (*cagA*, *vacA*, *iceA*), random amplified polymorphic DNA (RAPD), MLST, and whole-genome sequencing (WGS). WGS is the gold standard for research, but its application is limited due to its high cost and complexity of analysis (Wang and Gu, 2018). MLST is the standard method of molecular typing of bacteria because of its high resolution and repeatability (Raaf et al., 2017). In addition, it can be used to determine the genotype of ancestral strains in individual patients (Mendoza-Elizalde et al., 2019). Although RAPD is low cost and rapid, the PCR-based RAPD fingerprinting pattern has low reproducibility across different experiments (Yokota et al., 2015). Moreover, RAPD has limitations in determining *H. pylori* mixed infections: researchers can only subjectively determine whether there is relatedness between different strains by DNA fingerprinting and cannot determine the evolutionary relationships of these isolates from a single host. It is unclear whether strain diversity is a result of multiple-strain infections or microevolution occurring in a single ancestral strain. In this study, we used MLST to explore the occurrence of strain diversity. We concatenated seven housekeeping genes of each *H. pylori* strain and used the PHYLOViZ and neighbor-joining algorithm to show the evolutionary relationships among the strains. We found that most of the colonized strains of patients with mixed infections differed by only one housekeeping gene, suggesting that different genotypes may originate from a single ancestral strain. The evolutionary relatedness of strains can be identified by comparing STs (Mendoza-Elizalde et al., 2019), and in this study, five patients had more than three different housekeeping genes between their respective STs, which indicated that they were infected with unrelated strains. Moreover, four of these patients had isolates with seven different housekeeping genes, indicating infection with completely unrelated strains. The prevalence of *H. pylori* mixed infections involving completely unrelated strains was non-negligible. Notably, we found that unrelated *H. pylori* strains could colonize the same gastric site (corpus) or different sites (antrum and corpus), indicating that both the gastric antrum and corpus can be colonized by mixed unrelated strains, and that the same site can be infected with multiple different types of *H. pylori*. Previous studies have revealed the existence of a microevolutionary mechanism through slightly different DNA fingerprinting patterns among *H. pylori* isolates from a single host (Wong et al., 2001; Kim et al., 2003; Carroll et al., 2004; Farzi et al., 2019), and our study further revealed a mechanism of mixed infections based on MLST technology.

The prevalence of *H. pylori* mixed infections varies widely according to region. For example, the prevalence of mixed infections in developed countries/regions is generally lower than that in developing countries/regions (Ben Mansour et al., 2016). In this study, we used MLST and determined that the prevalence of *H. pylori* mixed infections among patients in Ningbo, China, was 41%, which is higher than that reported in other regions of China, such as Hong Kong (24% by RAPD) (Wong et al., 2001) and Taiwan (28.6% by detection of virulence genes) (Lai et al., 2016), but lower than that in Guiyang (76.9% by RAPD) (Mi et al., 2021). It remains to be investigated whether the differences in prevalence are related to the measurement method, other than the geographic region. This study confirmed that MLST is more reliable than RAPD for determining the prevalence of mixed infections. In this study, 12 different STs were obtained by MLST from *H. pylori* isolates isolated from 6 unrelated patient control groups and 6 ATCC controls, whereas only two different profiles from the clinical isolates were obtained by RAPD (data not shown), indicating that four isolates were not distinguished as different based on the RAPD type. However, almost all studies on mixed infections have not included controls and reference strains for *H. pylori* isolates from patients and ATCC strains (Wong et al., 2001; Kim et al., 2003; Carroll et al., 2004; Kao et al., 2014; Ben Mansour et al., 2016; Lai et al., 2016; Seo et al., 2019; Palau et al., 2020; Mi et al., 2021).

It is unclear whether the prevalence of *H. pylori* mixed infections may varies according to the presence of disease, and sex. The prevalence of mixed infections in duodenal ulcer patients was higher than other diseases (Lai et al., 2016), but other studies did not support this conclusion (Kim et al., 2004; Ben Mansour et al., 2016). One report suggested that the prevalence of *H. pylori* mixed infections was significantly higher in women than in men (Kibria et al., 2015), but other reports confirmed that the prevalence was unrelated to sex (Ben Mansour et al., 2016). The data from the Supplementary Table 1 showed that, in this study, the prevalence of *H. pylori* mixed infection was independent of disease status or sex. Whether these inconsistent findings are caused by different assay methods needs to be confirmed.

Reasons for the variation in the prevalence of mixed infections may also include the effects of the sampling strategies, such as biopsy specimens from one vs. several gastrointestinal sites and one vs. multiple colonies from each biopsy. The assay using *H. pylori* only from the antrum may not be representative of *H. pylori* populations in the entire stomach, and Wong et al. (2001) suggested that several different sites need to be assayed. In this study, we used *H. pylori* isolates from the gastric antrum, corpus, and/or duodenum bulb for the assays.

In this study, we identified 246 new alleles and 53 new STs associated with *H. pylori* infection. *H. pylori* is a highly genetically diverse species (Suerbaum et al., 1998). Analysis of the polymorphisms of housekeeping genes of *H. pylori* obtained from patients with mixed infections showed higher Hd values, which indicated that haplotypes forming the populations in each patient were very divergent, thus confirming the high diversity of *H. pylori*. The number of polymorphic sites revealed that *H. pylori* can mutate, making *H. pylori* more adaptable to specific ecological niches in individual hosts (Kang and Blaser, 2006; Naito et al., 2006).

Phylogenetic analyses of the seven concatenated housekeeping genes revealed different geographical groups of *H. pylori*. In this study, *H. pylori* was most prominently represented by the hspEAsia subgroup, and for the first time, three isolates (ST3699) recovered from a local patient with no history of living abroad were found to belong to the hpEurope group. MLST is a useful tool for tracking human migration (Yamaoka, 2009), enabling us to infer human migration routes or colonization history based on the geographic grouping of *H. pylori*. *H. pylori* strains that colonize in patients from Ningbo belong predominantly to the hspEAsia subgroup, which is consistent with the geographical location of Ningbo (eastern Asia).

In this study, we used a self-developed kit, Gu’s kit, for rapid isolation of *H. pylori*, which is a new technique for obtaining isolates rapidly and efficiently. Studies have reported that some genotypes were lost during culture passages (Ren et al., 2012) and eventually failed to obtain isolates (Arévalo-Jaimes et al., 2019), which is detrimental to the study of mixed infections. This new and rapid culture technique can effectively shorten culture time and reduce the loss of genetic representation during subculture passages, which can truly reflect the patient’s *H. pylori* mixed infections.

The main limitation of this study is the small sample size, which did not enable us to analyze the effect of the specific isolation scheme on the prevalence of mixed infections detected. Therefore, these results need to be confirmed by larger studies.

In conclusion, the prevalence of *H. pylori* mixed infections in patients from Ningbo, China, is high. Moreover, the prevalence of *H. pylori* mixed infections involving unrelated strains was 46%, and the prevalence of *H. pylori* mixed infections involving completely unrelated strains was 36%, which may be related to the use of new *H. pylori* isolation and culture techniques. In this study, 246 new alleles and 53 new STs were discovered for the first time. Most *H. pylori* strains in Ningbo belong to the hspEAsia subgroup, whereas very few strains belong to the hpEurope group.

## Data availability statement

The datasets presented in this study can be found in online repositories. The names of the repository/repositories and accession number(s) can be found in this article/Supplementary material.

## Ethics statement

The studies involving humans were approved by the Medical Research Committee of the Ningbo University School of Medicine. The studies were conducted in accordance with the local legislation and institutional requirements. The participants provided their written informed consent to participate in this study.

## Author contributions

HG conceived the study, collected gastrointestinal biopsy samples, and reviewed the manuscript. YZ performed the experiment, generated the sequence data, performed bioinformatics analysis, and prepared the manuscript. ZS, WC, WY, and CZ collected gastrointestinal biopsy samples. AL participated in the analysis of the results. MZ generated the sequence data. HY performed some of the experiments. All authors read and approved the final manuscript.

## Footnotes

1
http://pubmlst.org/helicobacter

2
https://pubmlst.org/organisms/helicobacter-pylori
