# Supplementary material for: High prevalence of Helicobacter pylori mixed infections identified by multilocus sequence typing in Ningbo, China
Source: Front Microbiol. 2023 Aug 8;14:1207878. doi: 10.3389/fmicb.2023.1207878 (PMC10442550; doi:10.3389/fmicb.2023.1207878)
Supplement: Supplementary file 1 [file Table_1.DOCX]

Additional file 1. Patients’ basic information and *Helicobacter pylori* isolates

| Patient | Age  (years) | Sex^1)^ | Diagnosis^2)^ | NHPI from ALBS^3)^ | | | Hospital^4)^ |
| --- | --- | --- | --- | --- | --- | --- | --- |
|  |  |  |  | A | C | DB |  |
| 1 | 62 | M | GU + DU | 0 | 0 | 1 | NF |
| 2 | 57 | M | GU | 0 | 0 | 1 | NF |
| 3 | 61 | F | GU | 1 | 0 | 0 | NF |
| 4 | 71 | M | CSG | 0 | 1 | 0 | NF |
| 5 | 31 | F | GU + DU | 1 | 0 | 0 | NF |
| 6 | 49 | F | CSG | 0 | 0 | 1 | NF |
| 7 | 30 | F | GU | 1 | 1 | 1 | NF |
| 8 | 34 | M | DU | 1 | 1 | 1 | NF |
| 9 | 49 | M | CAG | 0 | 1 | 1 | NF |
| 10 | 41 | M | DU | 1 | 1 | 1 | NF |
| 11 | 16 | M | CAG | 1 | 1 | 0 | NF |
| 12 | 59 | M | DU | 0 | 1 | 1 | NF |
| 13 | 41 | M | CAG | 1 | 1 | 1 | NF |
| 14 | 32 | F | CAG | 0 | 7 | 0 | NF |
| 15 | 50 | M | CAG | 0 | 0 | 8 | NF |
| 16 | 56 | M | GU | 1 | 2 | 1 | NF |
| 17 | 52 | M | CAG | 1 | 1 | 0 | NF |
| 18 | 44 | M | CSG | 1 | 1 | 1 | NF |
| 19 | 40 | F | CSG | 1 | 1 | 1 | NF |
| 20 | 28 | F | CSG | 1 | 1 | 1 | NF |
| 21 | 54 | M | CSG | 9 | 0 | 8 | NF |
| 22 | 65 | M | AG | 8 | 7 | 0 | NU |
| 23 | 39 | F | DU | 1 | 1 | 1 | NF |
| 24 | 56 | F | CAG | 1 | 1 | 1 | NF |
| 25 | 57 | F | CAG | 1 | 1 | 1 | NF |
| 26 | 51 | M | GU | 1 | 1 | 1 | NF |
| 27 | 32 | F | CAG | 1 | 1 | 0 | NF |
| 28 | 63 | F | CAG | 0 | 1 | 1 | NF |
| 29 | 63 | M | CAG | 1 | 1 | 0 | NF |
| 30 | 56 | F | CAG | 8 | 4 | 0 | NU |
| 31 | 70 | M | CSG | 8 | 8 | 0 | NU |
| 32 | 28 | F | GU | 5 | 0 | 0 | NU |
| 33 | 63 | F | CAG | 10 | 9 | 0 | NU |

1) Sex: M: Male, F: Female

2) Diagnosis: GU: gastritis ulcer, DU: duodenal ulcer, CSG: chronic superficial gastritis, CAG: chronic active gastritis, AG: atrophy gastritis

3) NHPI: number of *H. pylori* isolates, ALBS: anatomical location of biopsy specimen, A: antrum, C: corpus, DB: duodenal bulb

4) Hospital: NF: Ninghai First Hospital, NU: the Affiliated Hospital of Ningbo University School of Medicine
